# Microfluidic-LAMP chip for the point-of-care detection of gene-deleted and wild-type African swine fever viruses and other four swine pathogens

**DOI:** 10.3389/fvets.2023.1116352

**Published:** 2023-02-16

**Authors:** Chihai Ji, Ling Zhou, Yonghui Chen, Xueen Fang, Yanhong Liu, Mengkan Du, Xiandong Lu, Qianniu Li, Heng Wang, Yuan Sun, Tian Lan, Jingyun Ma

**Affiliations:** ^1^Guangdong Provincial Key Lab of Agro-Animal Genomics and Molecular Breeding, College of Animal Science, South China Agricultural University, Guangzhou, Guangdong, China; ^2^Guangdong Laboratory for Lingnan Modern Agriculture, College of Animal Science, South China Agricultural University, Guangzhou, China; ^3^African Swine Fever Regional Laboratory of China, College of Veterinary Medicine, South China Agricultural University, Guangzhou, China; ^4^Department of Chemistry and Institutes of Biomedical Sciences, Fudan University, Shanghai, China; ^5^Ningbo iGene Technology Co., Ltd., Ningbo, China; ^6^Hangzhou Xiaoshan District Animal Husbandry and Veterinary Development Center, Xiaoshan Bureau of Animal Husbandry and Veterinary, Hangzhou, China

**Keywords:** multiplex detection, microfluidic chip, loop-mediated isothermal amplification (LAMP), portable diagnostic tool, pathogens

## Abstract

**Introduction:**

Different pathogens causing mixed infection are now threatening the pig industry in the context of the African Swine Fever (ASF) circulating especially in China, and it is crucial to achieving the early diagnosis of these pathogens for disease control and prevention.

**Methods:**

Here we report the development of a rapid, portable, sensitive, high-throughput, and accurate microfluidic-LAMP chip detection system for simultaneous detection and differentiation of gene-deleted type and wild-type African swine fever virus (ASFV), pseudorabie virus (PRV), porcine parvovirus (PPV), porcine circovirus type 2 (PCV2), and porcine reproductive and respiratory syndrome (PRRSV).

**Results and discussion:**

The newly developed system was shown to be sensitive with detection limits of 101 copies/μl for ASFV-*MGF505-2R*/*P72*, PPV, and PCV2, 102 copies/μl for ASFV-*CD2v*, PRV, and PRRSV. The system was highly specific (100%) and stable (C.V.s < 5%) in its ability to detect different pathogens. A total 213 clinical samples and 15 ASFV nucleic acid samples were collected to assess the performance of the detection system, showing highly effective diagnosis. Altogether, the developed microfluidic-LAMP chip system provides a rapid, sensitive, high-throughput and portable diagnostic tool for the accurate detection of multiple swine pathogens.

## 1. Introduction

In recent decades, with the increasing demand for pork, the pig industry in China has witnessed a large-scale growth accompanied by multiple viral disease outbreaks (1). Under the intensive captivity conditions, it is common for sows to be infected simultaneously with a variety of pathogens such as African swine fever virus (ASFV), porcine reproductive and respiratory syndrome (PRRSV), porcine circovirus type 2 (PCV-2), porcine parvovirus (PPV), and pseudorabies virus (PRV) (2–4).

African swine fever (ASF) is a infections disease caused by ASFV with the mortality rates near 100% (5). Since the identification of ASF in China in August 2018, ASFV has been causing series of outbreaks which resulted in significant economic loss nationwide (6). Recently, newly emerging virulent mutant strains of ASFV with mutations, deletions, insertions, or short-fragment replacement in genomes have made the early diagnosis of ASF more difficult (7). Although artificial deletion of *EP402R* gene encoding the *CD2v* protein and multigene family 505 (*MGF505*) have been demonstrated to result in attenuated virulence in HLJ/18 strain and Georgia2007 isolate (8, 9), but their safety remains to be elucidated (10, 11). There is a need to identify the gene-deletion strain from the natural isolates, as the obtained phenotype of the genetically modified ASFV has some degree of uncertainty (12). This is essential to establish biosafety control measures in pig farms.

Porcine reproductive and respiratory syndrome (PRRS) is one of the most important economically diseases to the pig industry, which causes great losses to the pig industry in China (13). Pseudorabies (PR) caused by suid herpesvirus 1, is an economically important viral disease of pigs with high prevalence of its variants in China (14). PPV is one of the major etiologic agents of reproductive disorders in China, Canada and many other countries (15). PCV-2 is a widespread, economically challenging pathogen in most pig-producing countries and it has co-infections with multiple diseases including ASF and PRRS (16). The aforementioned diseases appear with similar symptoms such fever, loss of appetite, and reproductive impairments which makes it hard to diagnose at an early stage.

So far, the pathogens of swine diseases are commonly detected by single-pathogen isolation and identification, serological test, PCR and ELISA. As the same time, there are numbers of methods for detection on the basis of laboratory diagnosis. All the above-mentioned methods have been used extensively due to advantages of the accuracy and high sensitivity. Despite these merits, most of these assays are intricate and limited by specific laboratory settings and large amounts of reagents that can't be used in remote areas. Therefore, simple, rapid, reliable, and multi-targeted portable point-of-care (POC) diagnostic tools of swine pathogens are in urgent need for early disease detection and the prevention of viral transmission (17). Centrifugal microfluidic lab-on-a-chip (LOC) is an integrated and miniaturized technology that has the ability to combine sample extraction, reactions and detection on a compact disc (CD) (18, 19). This advanced technology makes detection assays more efficient and time-saving, which is calculated for Point of Care Testing (POCT) in viral detection (20).

In the present study, we integrated the loop-mediated isothermal amplification (LAMP) into the CD-like microfluidic chip for rapid multiplex detection of five pathogens in portable device. Our test shows that, the entire analysis including sample preparation and detection can be completed in 60 min, demonstrating high feasibility for the accurate, sensitive and specific detection of diseases in livestock farms in remote areas.

## 2. Materials and methods

### 2.1. Clinical samples, nucleic acid extraction and qRT-PCR assays

Clinical serum samples and tissue samples were collected from pigs that were suspected to carry fever and reproductive disorder- associated pathogens from 2016 to 2017 in South China in accordance with the recommendations of National Standards for Laboratory Animals of the People's Republic of China (GB149258-2010). All samples were preserved at −80°C from the time of original receipt until use. Fifteen ASFV positive nucleic acid samples and 50 ASFV negative serum samples were detected. The positive nucleic acid samples were provided by African Swine Fever Regional Laboratory of China (Guangzhou), and the negative serum samples were collected by our laboratory in 2018.

Individual samples including spleens, lungs, lymph nodes, tonsils, brain tissues, nose swabs, and kidney tissues were mixed and ground into homogenates with phosphate buffered saline (PBS; 20% w/v), and frozen and thawed three times, then centrifuged for 10 min at 10, 000 g. Viral nucleic acid was extracted using the Magnetic Viral DNA/RNA Kit (iGeneTech, Ningbo, China) according to the manufacturer's specifications. Total extraction time was 15 min and the products were stored at −80°C until used.

The qRT-RCR detection of ASFV, PRV, PPV, PRRSV and PCV2 was carried out with ChamQ Universal SYBR qPCR Master Mix (Vazyme, China) according to the previously described methods (21–27).

### 2.2. Chips and microfluidic device

**Figure 1:**
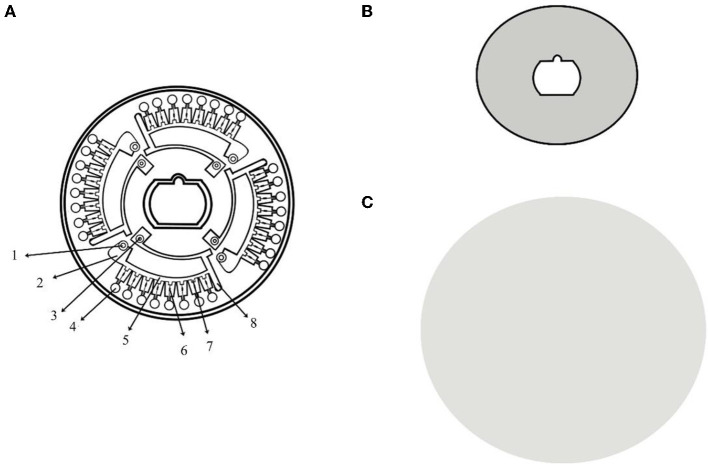
Structure chart of microfluidic chip. **(A)** The structure of the CD-like microfluidic chip. 1: sample well; 2: liquid storage chamber; 3: vent hole; 4: reaction chamber; 5: the second arc passage; 6: ball valve; 7: reservoirs; and 8: waste liquid tank; **(B)** Heat-sealing film of the front side; **(C)** Heat-sealing film of the back side.

The microfluidic chip used in this study were manufactured out of PMMA using micro-injection molding technology with a diameter of 80 mm and a thickness of 2.5 mm. A kind of heat-sealing film (SC-MA2000, Suchuang Co., Ltd) was used to form an integrated chip. Four independent units compose a microfluidic chip, each of which consisted of a sample well, a vent and eight micro reaction wells (Figure 1).

The ball valve played a pivotal role in the entire process ensuring the reaction mixture diffuse into the adjacent pipe and the reaction wells were filled with liquid. The volume of each micro reaction well was 5 μl. The supporting microfluidic detection appliance (dimensions: length, width, and height are 280 × 200 × 135 mm) was actually a device (Product Model: AJYGene TM MA2000) equipped with the functions of constant temperature control, high-speed centrifugation and fluorescence reading and analysis, which was provided by iGeneTech (Ningbo, China). The mixture containing the sample would be equably distributed in each reaction chambers at centrifugal speeds (1,600 rpm/min, 30 s; 4,600 rpm/min, 10 s) when the microfluidic device started running. Fluorescent signals were collected every minute for each reaction well and displayed in real time on the electronic screen during the reaction period.

### 2.3. Primer design

The reference complete genomic sequences of ASFV, PRV, PPV, PRRSV, and PCV2 were acquired from GenBank with accession numbers of MK333180.1, MH766894.2, KU056477.1, JQ249927.1, KU131565.1, and AF201897.1, respectively. To identify the gene-deleted type and wild type ASFV, we choose three genes (*CD2v, MGF505-2R*, and *P72*) as the target for the detection of ASFV. *CD2v* and *MGF505-2R* would not be detected if the sample contained gene-deleted type ASFV. The specific primers were designed based on the conserved nucleic acid fragments of ASFV *CD2v*/*MGF505-2R*/*P72*, PRV *gB*, PPV *VP2*, PCV2 *ORF2*, and PRRSV *P83* genes and synthesized by Beijing Genomics institution, BGI (Shenzhen, China). To assure the success of obtaining optimal primer sets, three sets of primers were designed for each gene. All primer sequences were listed in Table 1.

**Table 1:** Primer sequences used in microfluidic-LAMP chip system.

**Target**	**Primer**	**Sequence 5^′^-3^′^**
ASFV-*CD2v*	F3	GCTTCTTTGAAAGATGGACG
	B3	CTCATTTTTCGTTTGAATGACAA
	FIP	CGGGCCTTCTACAAAAAAATAAAGT-ATAAAAATCGCCAAAAAATGCTG
	BIP	TAATCACGTTGCCTATGCAAGC-TATGATCTCTAACCATAAGATGCG
	LB	TGACAGTTGGTTTGTTCTCGC
	ASFV-*MGF505-2R*	F3	AGGGTGATCAATATGACCTG
B3		TCTTCTTGGTATTTTTGGAGAA
FIP		CGCGTCTTGAATCAATGGTAAGA-ATCCATAAGTATGAAAACCAAATCG
BIP		ATACGTTTGAAAAATGCCACGC-AGCATGTTGTATTTTGTAGCA
LB		CGTTTTTGTGGTGTTTCATGTCTG
ASFV-*P72*		F3	ACTACTGCGAATACCCCGG
	B3	CGGGTTGGTATGGCTGC
	FIP	CCTGGCCGACCAAGTGCTTATGTTCGGATGTCACAACGCT
	BIP	TGGAGGGAACTAGTGGCCCT-ATAGGTTTGCTTTGGTGCGG
	LB	CTGGGATGCAAAATTTGCGC
	PRV-*gB*	F3	CCGTGCTCTTCAAGGAGAAC
B3		GCCTTGGAGACGCACTTG
FIP		GACCACACGGTCGTGACGATG-ATCGCCCCGCACAAGT
BIP		TACGCGGCCATCACGAACC-CGTCCGTGATCTCCTGCA
LF		GTTCTTGTAGTAGATGTGGGCC
PCV-2-*ORF2*		F3	TCTCATCATGTCCACCGC
	B3	CGGAGAAGAAGACACCGC
	FIP	CTTCAACACCCGCCTCTCCC-GGAGGGCGTTTTGACTGT
	BIP	TGCCATTTTTCCTTCTCCAGCG-CCATCTTGGCCAGATCCTC
	LB	CTTCGGATATACTATCAAGCGAACC
	PPV-*VP2*	F3	ATGGTCGCACTAGACACC
B3		ACTGTGTAGTCCTGTTTGT
FIP		GGTAACCATGGATAAAAACCAAGTGATAACACACTTCCATACACACC
BIP		ATTACCTATCATGCACCAGAAACCTTCTGTTATTTGTTCTGATTGTCC
LF		TTTCACTTCTAGGTGCTGC
PRRSV-2-*P83*		F3	GAAAGAAGGGGGATGGCC
	B3	CTGGATTGACGACAGACACA
	FIP	GCCTCTGGACTGGTTTTGCTGA-CCAGTCAATCAGCTGTGCC
	BIP	AACCCGGAGAAGCCCCATTTT-CGCTCACTAGGGGTAAAGTG
	LB	GCGACTGAAGATGATGTCAGACAT

### 2.4. Preparation of standard plasmids

All target gene fragments were synthesized and constructed into pMD19-T vector by BGI (Shenzhen, China), then were transformed into DH5α Escherichia coli cells following the manufacture's recommendations (TaKaRa, Dalian, China) and purified by Plasmid Mini Kit 2 (Omega Bio-tek, USA). The standard plasmids were tested and verified, and sent to BGI (Shenzhen, China) for sequencing. The concentration of the reconstructed standard plasmids was tested by a Qubit Fluorometer.

### 2.5. Setup of the microfluidic-LAMP system

**Figure 2:**
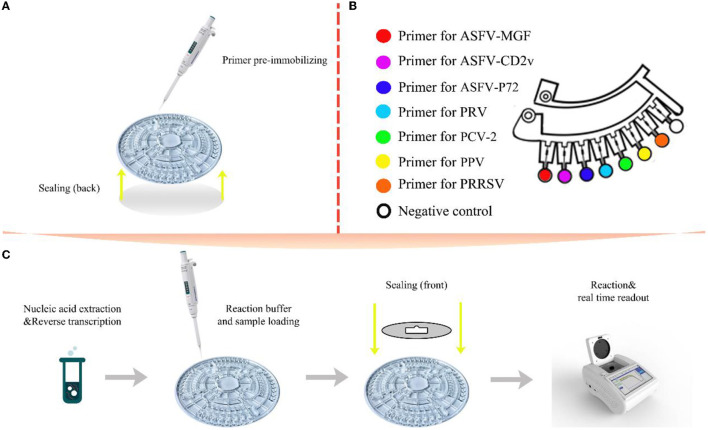
Schematic illustration of the multiplex detection based on microfluidic-LAMP chip system. **(A)** Primer pre-immobilizing and back side sealing; **(B)** a single section of the chip with its reaction area; **(C)** the entire flow of the diagnosis on microfluidic-LAMP chip detection system.

A volume of 5 μl primer mixture contains 0.2 μM each of outer primer (F3, B3), 1.6 μM each of inner primer (FIP, BIP), 0.8 μM each of loop primer (LF, LB), 0.3 μl trehalose and 1 μl ultrapure water. Before the start of the reaction, primer mixture was pre-immobilized in corresponding reaction chambers by heating drying. Seven micro chambers were activated for detections of seven viruses, and the remaining one was used for negative controls (Figure 2). Fifteen microliter of nucleic acid template and 10 μl premixed real-time LAMP reaction solution were transferred into the sample well by pipette and then sample wells and vent wells were sealed by the film. The preprocessed microfluidic chip was then placed in the provided microfluidic device reacted at 63.5°C for 60 min. Generally, the amplification result was regarded as positive when the chamber showed a positive signal and the negative controls showed negative signals in 40 min. A complete set of procedure was showed in Figure 2.

### 2.6. System evaluation and validation

#### 2.6.1. Specificity test

Viruses in this study including PRV, PRRSV, PPV, PCV2, and four other porcine viruses containing Classical swine fever virus (CSFV), foot and mouth disease virus (FMDV), seneca valley virus (SVA), and rotavirus (RV) were applied to verify the specificity of the assays. RNase Free ddH_2_O was also contained in the run as negative control.

#### 2.6.2. Sensitivity test

All the standard plasmids were diluted serially 10-fold with RNase free ddH_2_O (10^6^-10^0^ copies/μl) as templates to define the detection limit of every amplified system, and each detection limit test was repeated three times.

#### 2.6.3. Stability and reproducibility analysis

For stability verification, 10-fold serial plasmids dilutions with high (10^6^ copies/μl), medium (10^4^ copies/μl) and low (detection limits of each microfluidic LAMP chip system) concentrations were used to assess coefficients of variation (C.V.) of the microfluidic-LAMP chip system. Synthetic ASFV DNAs and clinical positive samples of PRV, PPV, PCV-2 and PRRSV were also applied to evaluate the reproducibility of the microfluidic-LAMP chip system. The C.V. was calculated by testing microfluidic-LAMP chip system in three consecutive runs (inter-group) in different chips and four times in the same chip (intra-group). Threshold time (Tt) was estimated from the reaction time when the positive signals of a particular sample exceed the baseline during the real-time amplification.

### 2.7. Clinical sample determination

A total of 163 swine clinical serum and tissue samples with fever and reproductive disorders, 15 ASFV positive nucleic acid samples and 50 ASFV negative serum samples were detected using the microfluid-LAMP chip system, and the diagnostic accuracy of the developed system was compared with the qRT-PCR assays.

## 3. Results and discussion

### 3.1. Specificity and sensitivity of the microfluidic-LAMP chip system

**Figure 3:**
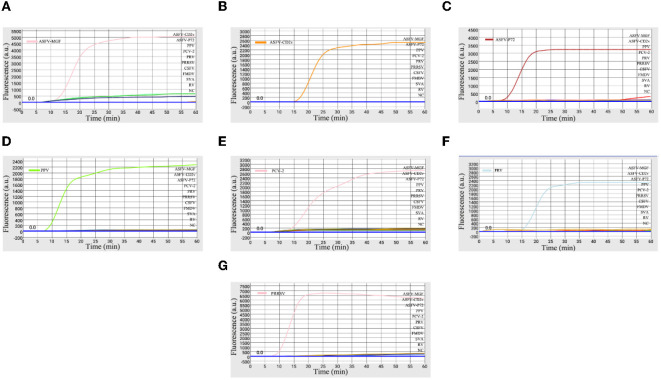
Specificity of microfluidic-LAMP chip detection system. **(A)** Specificity analysis for primers targeting to ASFV-*MGF505-2R*; **(B)** specificity analysis for primers targeting to ASFV-*CD2v*; **(C)** specificity analysis for ASFV-*P72*; **(D)** specificity analysis for PPV; **(E)** specificity analysis for PCV-2; **(F)** specificity analysis for PRV; and **(G)** specificity analysis for PRRSV.

The specificity of the microfluidic-LAMP chip system was determined with synthetic ASFV DNAs and viral nucleic acid including PRV, PPV, PRRSV, PRRSV, PCV2, CSFV, FMDV, SVA, and RV. As shown in Figure 3, only ASFV-MGF-, ASFV-*CD2v*-, ASFV-*P72*-, PRV-, PPV-, PCV2-, PRRSV-positive samples presented obvious amplification curves. Four other pathogens and the negative control did not show amplification curves, and no cross-reaction was observed during the amplification.

So far, there is no safe, effective and approved ASF vaccines, and only live attenuated ASF vaccines developed through gene-deletions are the most promising vaccine candidate in the near future (21). *CD2v* and *MGF505* genes have been considered as factors to reduce the ability of replication of the ASFV and have already been selected as the deletion target in live attenuated vaccines (8, 28). Thus, the identification of gene-deled type and wild type ASFV and other reproductive failure-associated swine pathogens including PRV, PPV, PCV2, and PRRSV becomes essential. Taken together, our system here displayed a high degree of specificity to the aimful viruses.

**Figure 4:**
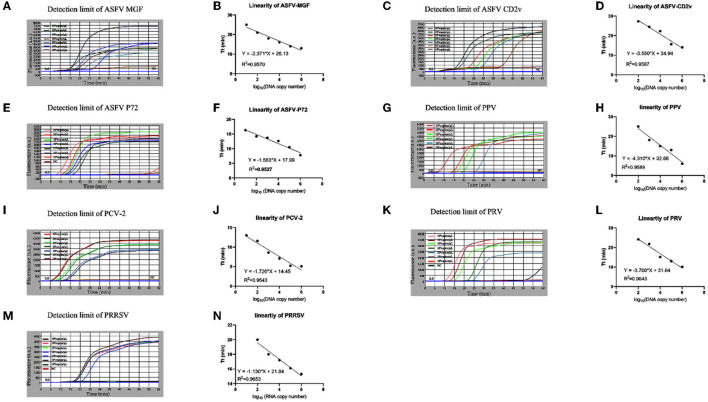
Sensitivity test of the microfluidic-LAMP chip detection system for five swine pathogens. **(A–N)** All tests were carried out using gradient-diluted standard plasmid of each pathogen. Semi-logarithmic regression between the Tt values and standards concentration by GraphPad Prism 8.3.0.

Serially diluted standard plasmids each with highly conserved genes of ASFV, PRV, PPV, PCV2, and PRRSV were used to investigate the detection limits of the microfluidic-LAMP chip system for those viruses. The results in Figure 4 showed that for ASFV-MGF/*P72*, PPV, and PCV-2 the detection limit of the established system was 10^1^ copies/μl, and for ASFV-*CD2v*, PRV and PRRSV the detection limit was 10^2^ copies/μl. The sensitivity analysis showed that the detection limit of each microfluidic-LAMP chip system was in the range of 10^2^ to 10^1^ copies/μl, which can be comparable to the existing detection methods (29–38). Based on the data of the sensitivity test with standard plasmids, the semi-logarithmic regression curves were established (Figure 4). The *R*^2^ values for ASFV-MGF, ASFV-*CD2v*, ASFV-*P72*, PRV, PPV, PCV-2, and PRRSV were 0.9570, 0.9587, 0.9527, 0.9589, 0.9543, 0.9643, and 0.9653, respectively and a significant linear relationship can be seen in each test, which indicate that the microfluidic- LAMP chip system has the potential for the quantitative detection.

### 3.2. Stability and repeatability of the microfluidic-LAMP chip system

**Figure 5:**
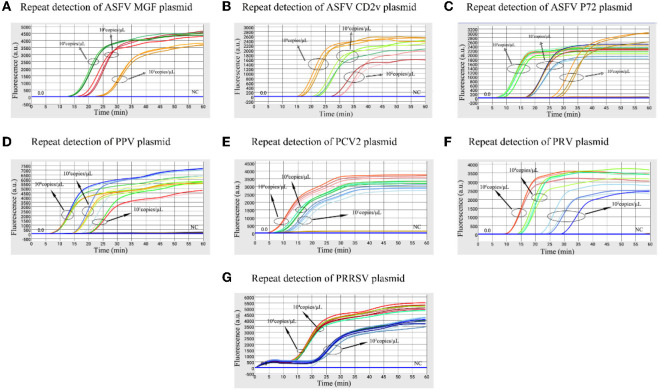
Stability test of the microfluidic-LAMP chip detection system for five swine pathogens. **(A–G)** All tests were performed using high, medium and low (detection limits) concentration standard plasmids.

**Figure 6:**
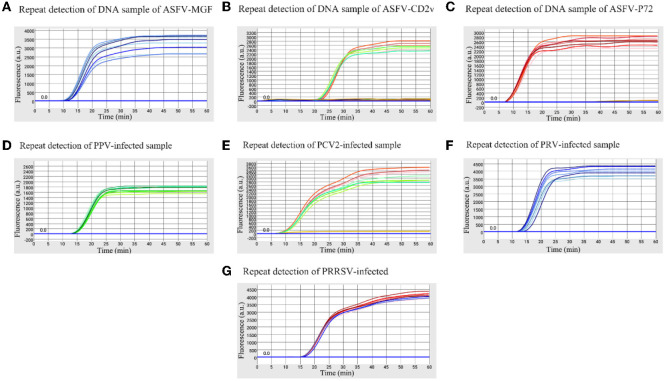
Reproducibility of the microfluidic-LAMP chip detection system for five swine pathogens. **(A–C)** Stability test of ASFV-MGF, ASFV-*CD2v*, and ASFV-*P72* for eight replicate experiments using a DNA sample. **(D–G)** Stability test of PRV, PPV, PCV-2, and PRRSV for eight replicate experiments using a clinical virus-infected sample.

The high, medium and low detectable concentration standard plasmids were used to test in triplicate to assess the stability of the microfluidic-LAMP chip system (Figure 5). There was a clear gradient from high to low plasmid concentration group of each test, which demonstrated the stability of the established method. To further assess the results, synthetic ASFV DNAs and the nucleic acid extracted from clinical positive samples of PRV, PCV2, PRRSV, and PPV were used for evaluation (Figure 6), which showed the good reproducibility for the developed system. All the C.V. values for the stability and reproducibility tests were < 5% (Table 2), indicating that the microfluidic-LAMP chip system has good reproducibility.

**Table 2:** The SD and C.V. of Tt values for each of eight replicate tests.

**Plasmid**	**Copy number (copies/μl)**	**Coefficient of variation (C.V.) (%)**	
		**Inter-group**	**Intra-group**
ASFV-*MGF505-2R*	×10^6^	1.73%	1.10%
	×10^4^	1.93%	1.44%
	×10^1^	1.00%	1.50%
	Nucleic acid sample	3.70%	3.01%
ASFV-*CD2v*	×10^6^	3.18%	2.15%
	×10^4^	2.98%	3.10%
	×10^2^	2.16%	2.69%
	Nucleic acid sample	1.76%	2.26%
ASFV-*P72*	×10^6^	2.33%	1.49%
	×10^4^	1.32%	0.62%
	×10^1^	3.96%	2.91%
	Nucleic acid sample	0.62%	1.08%
PRV	×10^6^	1.30%	2.54%
	×10^4^	2.08%	3.10%
	×10^1^	4.93%	4.96%
	Nucleic acid sample	1.48%	0.7%
PPV	×10^6^	3.6%	4.38%
	×10^4^	2.08%	3.61%
	×10^2^	4.52%	4.57%
	Nucleic acid sample	1.48%	2.73%
PCV-2	×10^6^	1.56%	1.84%
	×10^4^	1.61%	0.78%
	×10^1^	4.81%	4.45%
	Nucleic acid sample	2.11%	1.17%
PRRSV	×10^6^	1.39%	2.30%
	×10^4^	2.47%	0.96%
	×10^1^	1.11%	1.84%
	Nucleic acid sample	2.07%	2.89%

To compared the detection performances of the microfluidic chip system and qRT-PCR assays, 213 clinical serum samples and tissue samples (spleens, lungs, lymph nodes, tonsils, brain tissues, nose swabs and kidney tissues) and 15 ASFV positive nucleic acid samples were detected by microfluidic-LAMP chip system and qRT-PCR assay, respectively (Table 3). Testing of clinical samples with the microfluidic-LAMP chip system showed that, among the 146 serum samples, eight samples were positive for PCV-2 and 38 were positive for PRRSV, and there were no co-infections with PCV-2 and PRRSV. Among the 34 lung tissues samples, 14 were positive for PRV and two were positive for PCV-2 and there was no co-infection observed in these 16 PRV and PCV-2 samples. Testing of 10 tonsil tissues showed that six were positive for PRV, one was positive for PCV-2 and one was co-infected with PRV and PCV-2. PRV was detected in remaining 23 samples, accounting for 2 (2/7), 2 (2/3), 3 (3/8), 1 (1/5) of lymph nodes, brain tissues, nose swabs and kidney tissues, respectively. Fifteen nucleic acid samples tested positive for ASFV, no samples had confirmed PPV infection. Of these 92/228 (40.4%) tested positive by the microfluidic-LAMP chip system, which was slightly lower compared to 94/228 (41.2%) positive samples detected by the existing qRT-PCR assay. Except for the PRRSV (95%), the coincidence rate of the detection results of ASFV, PRV, PPV, and PCV-2 between the microfluidic-LAMP chip system and the existing qRT-PCR assay were all 100%. The clinical sample test revealed that PRV, PCV-2 ASFV, and PRRSV can be identified by the established system. The data suggested that the overall prevalence of positive samples was 40.4% (92/228), and PRV was detected in 29.3%, PCV-2 in 12% and PRRSV in 41.3% of the positive samples. The co-infection rate of PCV-2 and PRRSV was 8.7%. The positive detection rate of ASFV samples was 16.3%. The co-infection rate of PCV-2 and PRV was 3.3%. Despite no infection of PPV, these five viruses are still the common pathogens in swine production industry. Notably, the results for all of the 15 ASFV positive nucleic acid samples showed that an amplification curve could only be obtained for the *P72* channel, indicating that these positive samples were ASFV wild-type strains. This result was expected since the attenuated strains are not yet used as vaccines (Table 3). The coincidence rate of the detection results of PRV, ASFV, PPV, PCV-2, and PRRSV between the developed system and the existing qRT-PCR assay was 97.2%, indicating the new system can be applied to diagnosis of swine pathogens.

**Table 3:** Comparisons among the microfluidic- LAMP chip system from detection of PRV, PPV, PCV-2, ASFV, and PRRSV with the existing qRT-PCR assay using clinical samples.

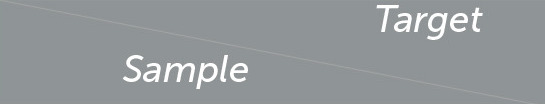		**ASFV-MGF**	**ASFV-*CD2v***	**ASFV*-P72***	**PRV**	**PPV**	**PCV-2**	**PRRSV**
Microfluidic-LAMP chip system	146 serum samples	0	0	0	0	0	8	38
	34 lung tissues	0	0	0	14	0	2	0
	10 tonsil tissues	0	0	0	6	0	1	0
	7 lymph nodes	0	0	0	2	0	0	0
	3 brain tissues	0	0	0	2	0	0	0
	8 nose swabs	0	0	0	3	0	0	0
	5 kidney tissues	0	0	0	1	0	0	0
	15 ASFV nucleic acid samples	15	15	15	0	0	0	0
qRT-PCR	146 serum samples	0	0	0	0	0	8	40
	34 lung tissues	0	0	0	14	0	2	0
	10 tonsil tissues	0	0	0	6	0	1	0
	7 lymph nodes	0	0	0	2	0	0	0
	3 brain tissues	0	0	0	2	0	0	0
	8 nose swabs	0	0	0	3	0	0	0
	5 kidney tissues	0	0	0	1	0	0	0
	15 ASFVnucleic acid samples	15	15	15	0	0	0	0

This study describes the development of a low-cost, rapid, field deployable, microfluidic-LAMP chip system for one-step-distribution of samples and real-time loop-mediated isothermal amplification, enabling the simultaneous detection and differentiation of ASFV, PRV, PPV, CSFV, and PCV-2 within 60 min. Pre-immobilization of different primers targeting to different pathogens allow for a broad range of microfluidic-LAMP chip purposefully customized for specific diagnosis, which has implications for clinical application for early detection of swine pathogens. The minimum equipment of our proposed system required only pipettes, reagent tubes, microfluidic chips and a portable detection device, which has great potential for clinical diagnosis outside of the laboratory.

## 4. Conclusions

Herein, we have developed a microfluidic-LAMP chip system for synchronous detection and identification of gene-deleted and wild-type ASFV, PRV, PPV, PCV2, and PRRSV on a single chip. The developed system demonstrates the detection limit in the range from 10^2^ to 10^1^ copies/μl, requires < 60 min for the entire analysis and is accurate in differential diagnosis of swine pathogens. Importantly, the microfluidic-LAMP chip system detection time was within 60 min, and the test results could be seen in 40 min at the fastest. The microfluidic-LAMP chip system possessed outstanding advantages in high-throughput, rapid detection, high sensitivity and simplified manipulation that shows its application potential of porcine pathogens diagnosis and control. This is crucial to the minute-by-minute battle in pandemic surveillance.

## Data availability statement

The original contributions presented in the study are included in the article/supplementary material, further inquiries can be directed to the corresponding authors.

## Ethics statement

The animal study was reviewed and approved by South China Agricultural University Experimental Animal Welfare Ethics Committee.

## Author contributions

Conceived and designed the experiments: JM, TL, and YS. Performed the experiments: CJ, LZ, YC, and XF. Sample collection: XF, YL, MD, XL, QL, and HW. Analyzed the data: CJ, LZ, YC, YS, and YL. Contributed to the writing: CJ and YC. All authors have read and approved the final manuscript.
